# Estimating Longitudinal Changes in Workday Happiness From Daily Report Texts Among Employees: Retrospective Feasibility Study

**DOI:** 10.2196/99992

**Published:** 2026-08-25

**Authors:** Junko Hayashi, Kazuhiro Ito, Masae Manabe, Yasushi Watanabe, Masataka Nakayama, Yukiko Uchida, Shoko Wakamiya, Eiji Aramaki

**Affiliations:** 1 Department of Information Science, Nara Institute of Science and Technology, 8916-5 Takayama-cho, Ikoma, Nara, 630-0192, Japan, 81 743-72-5250; 2 Department of Systems Innovation, School of Engineering, The University of Tokyo, Tokyo, Japan; 3 Office of Research Acceleration, Kyoto University, Kyoto, Japan; 4 Institute for the Future of Human Society, Kyoto University, Kyoto, Japan

**Keywords:** workday happiness, occupational health, occupational well-being, diary text, natural language processing, bidirectional encoder representations from transformers, BERT, large language models, longitudinal assessment

## Abstract

**Background:**

Workday happiness is associated with workplace performance and burnout, but frequent questionnaire-based assessment is burdensome in real-world workplace settings. Free-text daily reports may provide a lower-burden way to monitor day-to-day changes in workday happiness.

**Objective:**

This study aimed to examine whether daily diary text can be used to estimate longitudinal within-person changes in workday happiness and explore text-related factors associated with model performance, such as average sentence length and lexical diversity.

**Methods:**

We collected free-text daily reports and self-reported workday happiness scores from employees in 2 Japanese companies. Company A provided training data from 92 participants over 2 months (1725 reports), and company B provided test data from 11 participants over 6 months (652 reports). We used 2 text-based approaches: a bidirectional encoder representations from transformers (BERT)–based regression model trained on company A data and a locally deployed Japanese large language model (Llama) used in a zero-shot setting. Model performance was evaluated for each participant using the Pearson correlation coefficient between self-reported and estimated workday happiness scores, with *r*=0.40 used as a pragmatic feasibility benchmark. Mean absolute error (MAE) and root mean squared error (RMSE) were also calculated on the original workday happiness scale from 0 to 10.

**Results:**

A total of 81.8% (9/11) of the participants met or exceeded the feasibility benchmark of *r*=0.40. Positive correlations were observed for 81.8% (9/11) of the participants; for 9.1% (1/11) of the participants, the correlation coefficient could not be calculated because the self-reported score remained constant, and 9.1% (1/11) showed a small negative correlation. Participant-level correlations ranged from −0.06 to 0.65 for the BERT model and from −0.05 to 0.81 for the local large language model. Error-based metrics also varied across participants: BERT MAE ranged from 1.04 to 2.78, and RMSE ranged from 1.25 to 3.50, whereas Llama MAE ranged from 0.76 to 4.39, and RMSE ranged from 1.06 to 4.64.

**Conclusions:**

Our study suggests that daily report text supports low-burden, longitudinal estimation of individual-level workday happiness. However, performance varied across participants, and further work is needed to improve generalizability, reduce attrition, address possible measurement bias, and clarify appropriate workplace use.

## Introduction

Well-being, happiness, and mental quality of life (referred to hereafter as “happiness”) are key factors affecting workplace performance [1,2]. In the workplace, high levels of happiness are associated with better performance, greater cooperation [3], and greater career success [4]. Low happiness, in contrast, tends to cause problems such as decreased productivity [5]. As a result, the importance of longitudinal measurement of short-term happiness, such as day-to-day changes in the workplace, is increasing [6]. Monitoring happiness is therefore essential for enabling timely interventions such as encouraging employees to seek support when needed [7]. However, prior studies have primarily relied on questionnaires [8], which are not well-suited to capturing short-term changes in individual happiness in workplace settings. Most questionnaires, such as the Satisfaction With Life Scale [9], were not designed for daily assessment. In addition, questionnaires do not necessarily reveal what specifically made a person feel happy or what they perceived as the cause of their unhappiness.

Previous studies have demonstrated the feasibility of estimating happiness from text [10-16]. Early work in this area used naturally occurring social media language to predict individual or population-level well-being. For example, Schwartz et al [10] predicted individual well-being from social media language, whereas Jaidka et al [13] estimated geographic subjective well-being from Twitter data using both dictionary-based and data-driven language methods. Other research has explored linguistic indicators of well-being and happiness in social media and conversation texts, such as multilingual Twitter data and Echo (an application created by the study authors) text data [11,12]. More recent studies have used participant-generated free-text responses and machine learning methods to estimate subjective well-being more directly. Kjell et al [14] used bidirectional encoder representations from transformers (BERT) [17] to convert participants’ free-text responses into multidimensional vectors and predict individual questionnaire scores, suggesting that life satisfaction can be measured from free text with relatively high performance (*r*=0.74). Recent studies have also reported that large language models (LLMs) can estimate subjective happiness with high accuracy [16]. In workplace contexts, recent research has also applied sentiment analysis to employees’ open-ended or semi–open-ended responses to examine job satisfaction and identify workplace factors associated with positive or negative sentiment [18].

However, important gaps remain. Much of the previous work has focused on estimating cross-sectional questionnaire scores or global life satisfaction from free-text responses rather than estimating within-person longitudinal changes in happiness. In addition, less is known about whether routinely collected workplace daily reports can be used to estimate day-to-day fluctuations in workday happiness or whether a model trained in one organizational context can be applied to data from another company.

**Figure 1.**
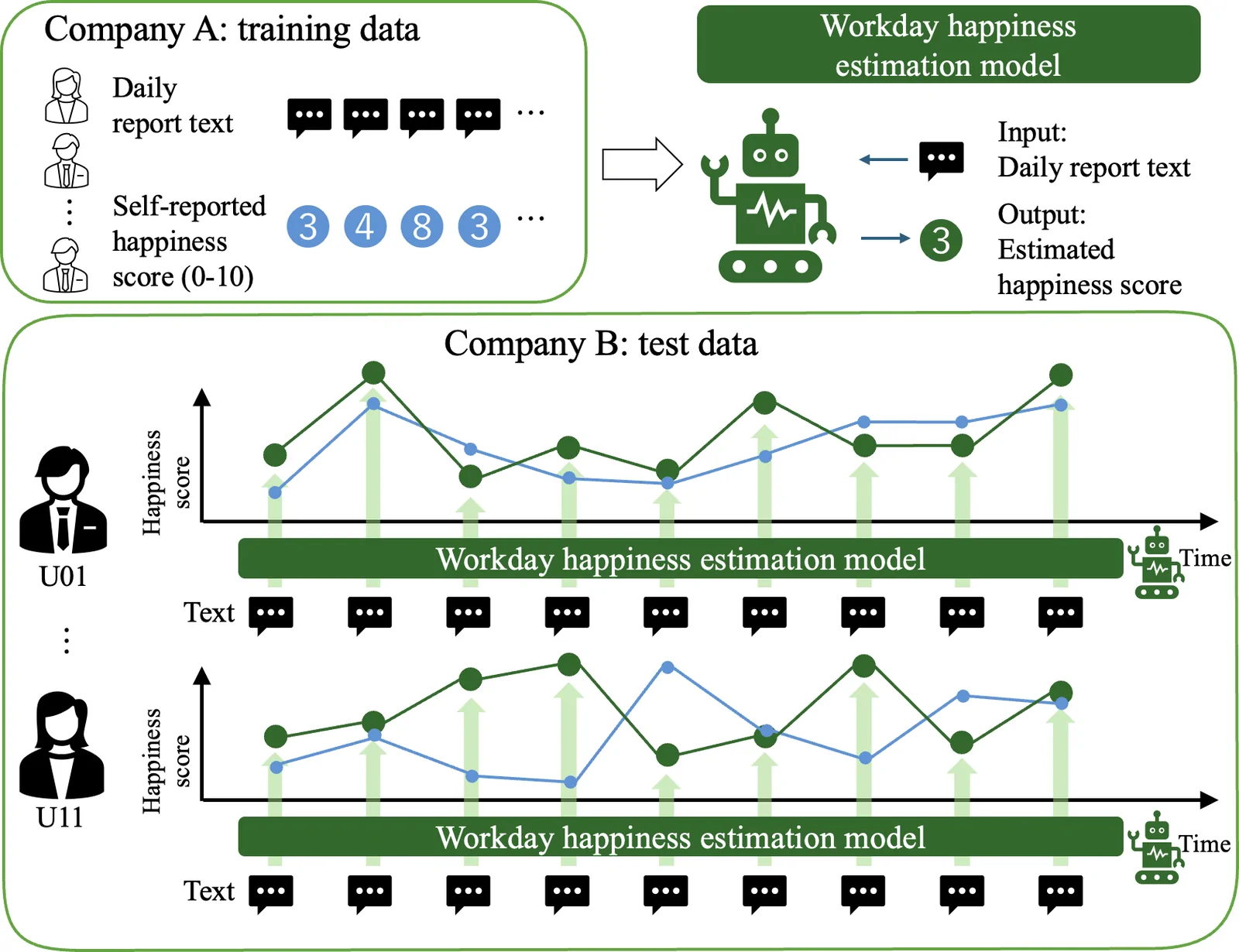
Overview of this study. To conduct short-term surveys that cannot be easily conducted using questionnaires, this study estimated changes in workday happiness levels based on daily report texts.

This study aimed to examine the preliminary feasibility of estimating within-person longitudinal changes in workday happiness from free-text daily reports using a BERT-based regression model and a locally deployed Japanese LLM (Figure 1). In this study, “workday happiness” refers to a daily evaluative happiness rating reported on workdays in a workplace context rather than global life happiness. We collected free-text daily report text together with self-reported workday happiness scores from employees in 2 workplaces.

This paper is an extended version of a previous paper [19]. The present study expands the prior work by adding new analyses based on the same dataset, including error-based evaluation metrics, exploratory analyses of diary characteristics, and a comparison with a locally deployed LLM in addition to the BERT-based model.

## Methods

### Study Design

This study was designed as a retrospective feasibility study using workplace daily report data collected from 2 Japanese companies. The company A dataset was used to train the BERT-based regression model, whereas the company B dataset was used as an external test dataset to evaluate whether the trained model and a locally deployed Llama model could estimate within-person longitudinal changes in workday happiness from daily report texts.

### Sample Size Rationale

Because this was a retrospective feasibility study based on existing workplace daily report datasets, the sample size was determined by the number of consenting employees who had available paired data consisting of daily report text and self-reported workday happiness scores during the data collection periods rather than by an a priori power calculation. The sample therefore reflected the available longitudinal workplace data from employees in the 2 participating Japanese companies. Participant and dataset characteristics are described in the Participants and Datasets section.

### Data Collection Procedure

We collected 2 types of data: daily report text and self-reported workday happiness scores. Daily reports consisted of free-text descriptions in Japanese of the participants’ daily lives with no character limit. Participants were instructed as follows (translated from Japanese): “Daily report text (required): Please write your diary in about 3 lines (it may be unrelated to work). Free description with no character limit.” Examples of the daily reports are provided in Table 1.

**Table 1.** Examples of daily report text and self-reported workday happiness scores.

Daily report text—translated from Japanese	Daily report text—Japanese	Self-reported workday happiness score (0-10)
“The most significant advantage of remote work is being able to nap. Although rather conspicuous, I would like to be able to nap in the office as well.”	リモート勤務の一番のメリットは, 昼寝できることだと思う。目立つけど, オフィスでも昼寝できたらいいのにな。	7
“Rushing on Monday mornings. Addressing complaints is difficult.”	月曜の朝はバタバタ。クレーム対応は難しい。	2
“Although I had much work to perform since morning, I participated in a fun drinking party! I enjoyed interacting with some people I had not met before. The food at the standing bar and the wine were delicious, and it was a great start to the week. It was incredibly fun.”	朝から仕事は多かったけど, 楽しい飲み会に参加した！初めて会う人とも話せてよかった。立ち飲みの料理もワインも美味しくて, 週の始まりとして最高。めちゃくちゃ楽しかった。	10
“Insufficient time... I am exhausted...”	時間が足りない……疲れた……	1
“I had new insights for marketing. Additionally, I am continuing with the accident response.”	マーケのことで新しい気づきがあった。あと, 引き続き事故対応。	5

Participants also rated their workday happiness on an 11-point scale ranging from 0 (extremely unhappy) to 10 (extremely happy). The item wording was as follows (translated from Japanese): “Were you happy today? 0 (very unhappy) to 10 (very happy).” This item was based on the ladder by Cantril [20].

Daily reports were collected using an original web browser–based application. Participants were instructed that entries should typically be submitted once or more per workday to reflect the events of that day at the end of the workday.

### Participants and Datasets

We used data from 2 companies. Company A is a major advertising and marketing company in Japan. In company A, 92 members submitted daily reports over 2 months from September 1, 2022, to October 31, 2022, yielding 1725 data points. The mean age of participants in company A was 39.0 (SD 9.0) years, and 22.8% (21/92) were female. Company B is a major Japanese electronics manufacturer. In company B, 11 employees submitted daily reports over 6 months from December 19, 2022, to May 18, 2023, yielding 652 data points. The mean age of participants in company B was 42.3 (SD 10.1) years, and 9.1% (1/11) were female. The average number of characters per entry was 58.8 in company A and 72.2 in company B (Table 2).

**Table 2.** Data statistics for companies A and B.

Company	Users, n	Reports, n	Characters per entry, mean (SD)	Period	Age (y), mean (SD)	Gender distribution (female), n/N (%)
A	92	1725	66.1 (49.3)	September 1, 2022, to October 31, 2022 (2 months)	39.0 (9.0)	21/92 (22.8)
B	11	652	75.0 (39.4)	December 19, 2022, to May 18, 2023 (6 months)	42.3 (10.1)	1/11 (9.1)

### Recruitment and Eligibility Criteria

Participants were employees of 2 Japanese companies that implemented daily workplace report systems during the study periods. Participants were recruited through workplace-based study invitations and provided informed consent before data collection. Eligible participants were employees who submitted daily report text entries with corresponding self-reported workday happiness scores during the data collection period. Reports were included in the analysis when both the daily report text and the self-reported workday happiness score were available. Reports with missing text, missing happiness scores, or invalid date information were excluded from the corresponding analyses.

### Model Development

#### Overview

In this study, we evaluated workday happiness estimation using 2 language model–based approaches: a BERT-based model and a locally deployed LLM. Both models took the daily report text as input and produced an estimated workday happiness score as output. The primary evaluation focused on how well the estimated scores tracked temporal fluctuations in self-reported workday happiness.

#### BERT-Based Model

We constructed a BERT-based estimation model in which the input was the daily report text and the output was the estimated workday happiness score. The training data were obtained from company A. Specifically, we built a regression model using AutoModelForSequenceClassification based on Tohoku University’s pretrained Japanese BERT [21]. The learning rate was set to 0.000002, the number of epochs was set to 20, and AdamW was used as the optimizer [22].

#### Local LLM

As the second model, we used a lightweight locally deployed LLM with enhanced Japanese-language capability. We chose a local LLM because transmitting workplace diary data to an external service may be undesirable in practical workplace settings; therefore, we examined a model that could run in a local environment. Specifically, we used tokyotech-llm’s Llama-3.1-Swallow-8B-Instruct-v0.5 [23,24], which is based on Meta AI’s Llama 3.1 (8B) [25]. Generation was conducted with greedy decoding, and the maximum number of new tokens was set to 8. To evaluate feasibility under strict conditions, we used the LLM in a zero-shot setting; that is, we did not include examples in the prompt and did not perform additional fine-tuning using workplace data. The prompt was as follows (translated from Japanese): “You are an evaluator who estimates the writer’s happiness from a diary entry. Please estimate the happiness of the writer of the following diary on a scale from 0 to 10. Output only 1 integer and no other characters.” The prompt was designed to be simple, task specific, and constrained to output a single integer between 0 and 10, matching the self-reported workday happiness scale. Because this initial evaluation focused on testing the locally deployed LLM with a simple prompt rather than optimizing performance through prompt engineering, we did not examine sensitivity to different prompt designs.

### Evaluation Metrics

The purpose of this study was not primarily to estimate the absolute level of workday happiness but rather to assess whether changes in workday happiness could be captured over time. Therefore, we used the Pearson correlation coefficient as the main evaluation metric following previous work [15]. We interpreted the Pearson correlation coefficient (*r*) as an effect size index indicating the degree to which self-reported and estimated scores increased and decreased in the same direction over time, that is, the extent of agreement in temporal fluctuation. Following prior work, we used *r*=0.40 as a pragmatic reference point for this feasibility evaluation rather than as a threshold for real-world workplace implementation [19]. To contextualize this value, we also reviewed prior work in personnel and applied psychology. For example, Schmidt and Hunter [26] reported that several commonly used predictors of job performance had validity coefficients in this range. Bosco et al [27] argued that correlations should be interpreted using empirical benchmarks from the relevant research context rather than generic rules of thumb. However, a moderate correlation suggests only that the model may capture some within-person temporal signal.

Because the Pearson correlation coefficient does not quantify the absolute magnitude of prediction errors, we additionally used the mean absolute error (MAE) and root mean squared error (RMSE) as error-based metrics. The MAE represented the average absolute difference between self-reported and estimated workday happiness scores, whereas the RMSE gave greater weight to larger prediction errors. Both MAE and RMSE were calculated on the original workday happiness scale from 0 to 10.

### Data Analysis

Model performance was evaluated at the participant level in the external test dataset from company B. For each participant and each model, we calculated the Pearson correlation coefficient between self-reported and estimated workday happiness scores. Pearson correlation tests were conducted as 2-tailed tests with a significance level of α=.05. To quantify uncertainty in the participant-level correlation estimates, we calculated 95% CIs for Pearson correlation coefficients using the Fisher *z* transformation. CIs were not calculated when the Pearson *r* was undefined because the self-reported workday happiness score was constant. We also calculated MAE and RMSE for each participant and each model to quantify absolute prediction errors on the original workday happiness scale from 0 to 10.

Participant-level MAE and RMSE values were compared between the BERT-based model and the Llama model using 2-tailed paired *t* tests, with statistical significance set at α=.05. Effect sizes for paired *t* tests were reported as Cohen *dz*. Days without diary entries or self-reported workday happiness scores were treated as missing and excluded from the corresponding participant-level analyses. No imputation was performed.

We also conducted exploratory participant-level analyses to describe whether diary text characteristics varied according to model performance. Specifically, we conducted 4 exploratory participant-level Pearson correlation analyses: BERT performance vs average sentence length, Llama performance vs average sentence length, BERT performance vs Self-BLEU, and Llama performance vs Self-BLEU. Average sentence length was calculated for each participant across daily report texts. Lexical diversity was evaluated using Self-BLEU [28]. A higher Self-BLEU value indicated that a participant’s daily reports were more similar to one another and, therefore, less lexically diverse, whereas a lower Self-BLEU value indicated greater lexical diversity. Self-BLEU was calculated using SacreBLEU [29]. Participant U01 was excluded from these exploratory analyses because model performance based on the Pearson correlation coefficient could not be calculated for them. For visualization only, participants were divided into below-median and above-median performance groups for each model in the box plots. These groups were not used to calculate separate correlations.

### Ethical Considerations

This study was approved by Kyoto University, including Nara Institute of Science and Technology (review 26-P-16). The company A and company B datasets used in this study were collected as part of a previous field study of daily workplace reports [30]. Before beginning the questionnaire, participants read an online explanation of the study overview and provided electronic informed consent.

To protect participant privacy and confidentiality, all datasets used in the present analysis were deidentified prior to analysis. Participants were instructed not to include personal information or company-confidential information in their free-text diary entries. In the present manuscript, participants in company B are referred to using pseudonymous labels (U01-U11), and no directly identifying information is reported. Because free-text daily reports may contain potentially identifiable information, raw text data are not publicly shared. Instead, only aggregated or statistically processed results are reported. Participation was voluntary. Participants did not receive financial compensation or other incentives. The model outputs in this study were analyzed retrospectively for research purposes and were not used to make employment, personnel, or managerial decisions about individual participants.

## Results

### User Statistics and Participant-Level Model Performance

**Figure 2.**
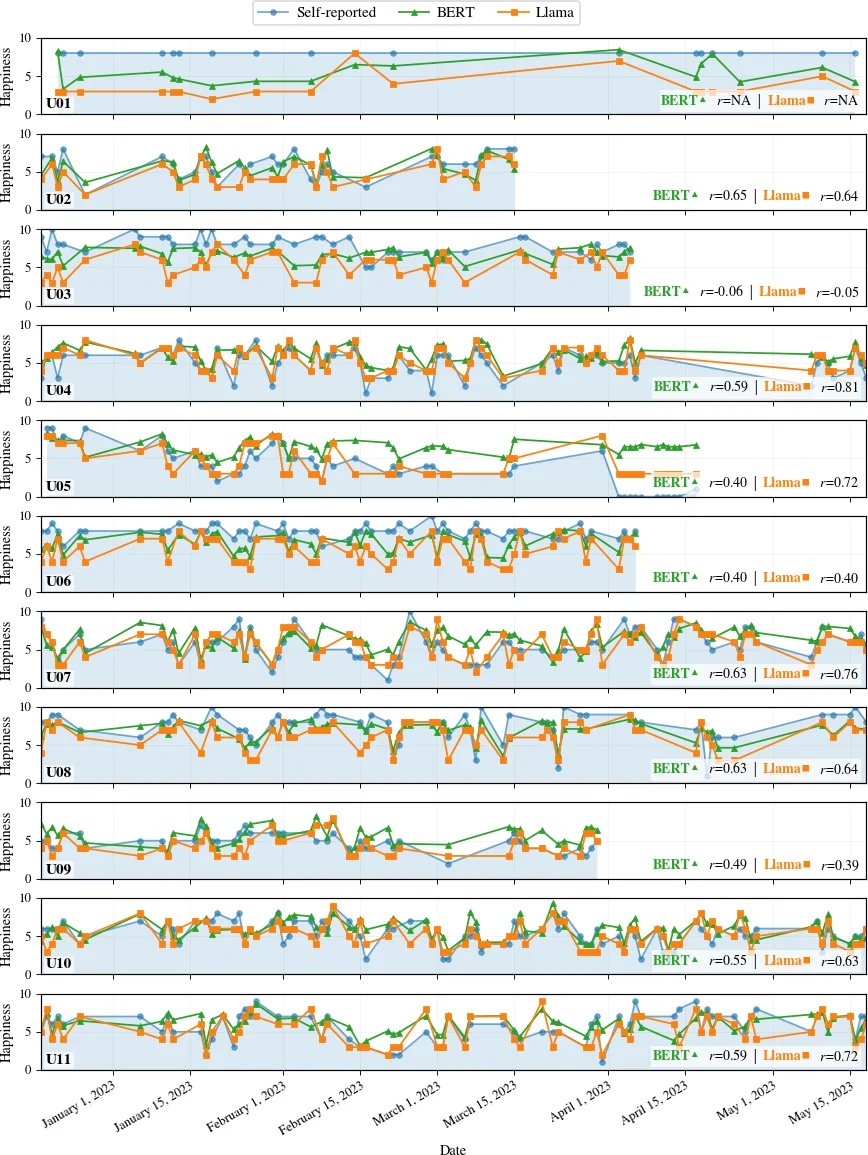
Time series of self-reported and estimated workday happiness scores for each participant in company B. The blue line indicates self-reported workday happiness scores, the green line indicates estimates from the bidirectional encoder representations from transformers (BERT)–based model, and the orange line indicates estimates from the locally deployed Llama model. Blank periods indicate days without diary entries. NA: not applicable.

Using *r*=0.40 as a pragmatic feasibility benchmark, 81.8% (9/11) of the users met or exceeded this level, indicating that the model achieved practically meaningful alignment for most users. The correlation coefficients of workday happiness estimation for each user in company B are shown in Table 3, and the time series are shown in Figure 2. As shown in Table 3, model performance varied across users. Hereafter, individual users are denoted as U01 to U11. Error-based metrics also showed variability across participants (Table S1 in Multimedia Appendix 1). For the BERT-based model, MAE ranged from 1.04 to 2.78, and RMSE ranged from 1.25 to 3.50 on the original workday happiness scale from 0 to 10. For the Llama model, MAE ranged from 0.76 to 4.39, and RMSE ranged from 1.06 to 4.64. The participant-level mean MAE and RMSE were 1.52 and 1.87 for the BERT-based model and 1.71 and 2.07 for the Llama model, respectively. As an exploratory comparison of absolute prediction error between models, paired *t* tests were conducted using participant-level MAE and RMSE values. No statistically significant difference was observed between the BERT-based model and the Llama model for MAE (*t*_10_=–0.72; *P*=.49; Cohen *dz*=–0.22) or RMSE (*t*_10_=–0.67; *P*=.52; Cohen *dz*=–0.20).

For U01, the correlation coefficient could not be calculated because the self-reported workday happiness score remained consistently at 8 (Figure 2 and Table 3). As shown in Figure 2, while users’ reported levels of happiness remained constant, the language model’s estimated levels of happiness showed some variation. Next, 9.1% (1/11) of the users showed a negative correlation coefficient, although it was not significant (BERT: *P*=.70; Llama: *P*=.72; Figure 2 and Table 3; U03). We examined the daily report text of this user and found that the user frequently used the expression “A nice day that...” As shown in Figure 2, only 1 user (U05) repeatedly self-reported a workday happiness score of 0 after April 2023. Although the user initially provided diverse texts, after April 2023, the entries were primarily zeros, and the content became uniform. In addition to this user, there were other users for whom the number of entries decreased since April 2023 (Figure 2; U2, U3, U5, U6, and U9).

In the comparison between models, Llama outperformed BERT for several users (U04, U05, U07, and U11), whereas BERT performed better for U09, and the 2 models showed similar performance for U06.

The 95% CIs indicated uncertainty in participant-level performance estimates. Although several point estimates met or exceeded the pragmatic feasibility reference point of *r*=0.40, some CI lower bounds were below this value.

**Table 3.** Participant-level correlations between self-reported and estimated workday happiness scores.

User ID	Daily reports, n	Self-reported workday happiness score (0-10), mean (SD)	BERT^a^-based model			Llama model		
			*r* (95% CI)	*r* ^2^	*P* value	*r* (95% CI)	*r* ^2^	*P* value
U01	18	8.00 (0.00)	—^b^	—	—	—	—	—
U02	35	5.89 (1.59)	0.65 (0.40 to 0.81)	0.42	<.001	0.64 (0.39 to 0.80)	0.41	<.001
U03	48	7.85 (1.22)	−0.06 (−0.34 to 0.23)	0.00	.70	−0.05 (−0.33 to 0.24)	0.00	.72
U04	75	5.05 (1.63)	0.59 (0.42 to 0.72)	0.34	<.001	0.81 (0.71 to 0.88)	0.66	<.001
U05	53	4.02 (2.67)	0.40 (0.15 to 0.60)	0.16	.003	0.72 (0.56 to 0.83)	0.51	<.001
U06	61	7.97 (0.75)	0.40 (0.16 to 0.59)	0.16	.002	0.40 (0.16 to 0.59)	0.16	.002
U07	87	5.61 (1.84)	0.63 (0.48 to 0.74)	0.40	<.001	0.76 (0.65 to 0.84)	0.58	<.001
U08	66	7.62 (1.85)	0.63 (0.46 to 0.76)	0.40	<.001	0.64 (0.47 to 0.76)	0.41	<.001
U09	47	4.81 (1.12)	0.49 (0.24 to 0.68)	0.24	<.001	0.39 (0.12 to 0.61)	0.15	.006
U10	91	5.42 (1.55)	0.55 (0.39 to 0.68)	0.31	<.001	0.63 (0.49 to 0.74)	0.40	<.001
U11	71	5.73 (1.88)	0.59 (0.41 to 0.72)	0.35	<.001	0.72 (0.58 to 0.82)	0.51	<.001

a BERT: bidirectional encoder representations from transformers.

b The correlation coefficient could not be calculated because the self-reported workday happiness score remained consistently at 8.

### Diary Characteristics and Model Performance

#### Overview

In this section, we present the exploratory results regarding the relationship between diary text characteristics and model performance. These analyses excluded U01 and were based on 10 participants; therefore, the findings were interpreted descriptively and as hypothesis generating only. The exploratory correlations were calculated across the 10 participants included in these analyses and were not calculated separately within the below-median and above-median performance groups.

#### Average Sentence Length

Users with longer average sentence lengths showed a descriptive tendency toward higher model performance. However, the pattern differed across models. The correlation between model performance and average sentence length was stronger for BERT (*r*=0.61; *P*=.06) than for Llama (*r*=0.33; *P*=.35), although neither correlation was statistically significant (Figure S1 in Multimedia Appendix 1). This result may be attributable to the limited sample size, the influence of outliers, or a nonlinear rather than monotonic relationship, suggesting that sentence length alone cannot fully account for performance differences.

#### Lexical Diversity

Participants with more lexically diverse daily reports showed a descriptive tendency toward higher model performance. However, the pattern differed across models. Self-BLEU showed a negative association with BERT performance (*r*=–0.51; *P*=.13), although this association was not statistically significant (Figure S2 in Multimedia Appendix 1). A similar but weaker pattern was observed for Llama (*r*=–0.15; *P*=.67). Given the small number of participants and the nonsignificant associations, these findings should be interpreted descriptively and as hypothesis generating only.

## Discussion

### Principal Findings

This study examined whether daily report texts could be used to estimate within-person longitudinal changes in workday happiness among Japanese employees. A BERT-based regression model trained on company A data and a locally deployed Llama model were evaluated using an external test dataset from company B. Overall, the findings suggest preliminary feasibility for capturing broad within-person temporal trends in workday happiness from daily report text. However, MAE and RMSE showed participant-level variation in prediction error, indicating that the findings should be interpreted cautiously and not as definitive validation.

### Interpretation and Implications

The findings suggest that daily report texts may contain information related to within-person changes in workday happiness. Open-ended responses may provide contextual information about work-related events or conditions that coincide with changes in self-reported workday happiness. For instance, if a decline in workday happiness is inferred and a respondent states, “I could not make any progress at work because the Wi-Fi did not connect,” this may indicate the need to investigate Wi-Fi connectivity issues. Similarly, a comment such as “The meeting was too long and I became fatigued” could serve as a catalyst for re-evaluating meeting durations. Thus, workday happiness estimation models may help identify recurring workplace issues that warrant further human review. However, the present findings should be interpreted as preliminary feasibility evidence rather than evidence that model outputs can directly identify the causes of changes in workday happiness or prescribe specific interventions.

Second, even when BERT-based models and LLMs are used as predictive models for workday happiness, their use in workplace settings requires careful consideration. Prediction performance alone is not sufficient to justify workplace deployment because the main issue is how estimated scores are interpreted and used in practice. If such estimates are treated as definitive judgments of employees or are used for personnel evaluation, they may undermine trust and discourage honest diary writing [31,32]. Therefore, predicted workday happiness scores should be used as supportive indicators for happiness interventions and workplace improvement rather than as direct evidence for employee assessment. In addition, because diary texts may contain sensitive personal information and model predictions may be biased or difficult to interpret, organizations need clear data handling policies.

Third, to collect daily report text over an extended period, it is necessary to mitigate participant dropout. Prior research also indicates that diary studies need to address dropout [33,34]. Several participants discontinued diary submission before the end of the observation period. This suggests that keeping a diary is burdensome, thereby posing a constant risk of attrition. Many participants stopped inputting their entries after April 2023, suggesting that environmental changes might have imposed stress that hindered their ability to continue diary writing (Figure 2). The potential solution involves using services that make it easier to write daily report texts. For example, a service could be provided to facilitate diary entry [35], or a service using LLMs to assist with writing could be used [36]. With these methods, the data collection rate may increase without requiring participants to be repeatedly prompted to submit diary entries.

Fourth, considering user-specific characteristics such as gender and age may improve the model’s performance in workday happiness estimation. Research on subjective descriptions of happiness collected through daily report text has demonstrated that a wide variety of descriptions are gathered [37]. What is considered workday happiness depends on attributes such as culture [38] and age [39]. In fact, taking into account age, gender, and personality has been shown to enhance model performance [40-43]. Therefore, collecting additional data through separate questionnaires may improve workday happiness estimation accuracy.

Finally, differences in model performance may reflect user-specific characteristics, including the length and content of daily reports, the explicitness of emotional expressions, and the types of work-related events described. A previous study comparing ChatGPT with fine-tuned BERT-style models reported that relative performance varied across natural language understanding tasks; ChatGPT performed worse on paraphrasing and similarity tasks but showed comparable or better performance on other tasks [44]. In the present study, model performance differences could also not be explained by text length alone. Other factors such as diary content, emotional clarity, individual writing style, and differences in the determinants of workday happiness may also have influenced model performance.

### Comparison With Prior Work

Previous studies have shown that happiness and subjective well-being can be estimated from text, including social media language, conversational text, free-text questionnaire responses, and LLM-based assessments [10-16]. For example, Kjell et al [14] reported relatively high performance when predicting questionnaire-based well-being scores from free-text responses using BERT-based representations. The present findings are consistent with the literature, suggesting that free-text language contains information related to self-reported happiness.

Unlike previous studies that estimated well-being from cross-sectional free-text questionnaire responses [14-16] or from social media language at the individual, population, or geographically aggregated level [10,13], the present study examined within-person longitudinal changes in workday happiness using routinely collected workplace daily reports. In addition, the BERT-based model was trained on data from one company and evaluated on data from another, yielding a more stringent preliminary evaluation than a random split within a single dataset.

### Limitations

This study has several limitations.

First, the relationship between specific events and workday happiness varies across individuals. For example, the statement “Today I have a meeting!!!” may evoke workday happiness for some individuals but not for others. Understanding the relationship between holidays and workday happiness requires not only sufficient textual data but also extensive information about the writers’ beliefs, values, and current circumstances.

Second, the generalizability of the model is limited. The model used in this study was based on data collected from experiments conducted by two companies: company A, an advertising and marketing firm, and company B, an electronics manufacturer. In addition, expressions of workday happiness may be influenced not only by industry but also by occupation, organizational culture, daily report writing practices, and cultural background. Therefore, the present findings may not directly generalize to nonoffice workers, smaller companies, or non-Japanese workplace settings.

Third, the daily report text collected may have been influenced by the workday happiness scores collected at the same time. Prior research on affect labeling [45] has shown that putting feelings into words can attenuate emotional experience and may function as a form of implicit emotion regulation. Future studies should adopt a design that separates the collection of free-form diary entries from self-reported happiness assessments.

Fourth, the small external test sample limits the statistical robustness and generalizability of the findings. Although repeated-measure datasets can be useful for initial feasibility analyses of within-person variability in related fields [46,47], the company B dataset included reports from only 11 participants, and the exploratory analyses of diary characteristics were based on 10 (90.9%) participants after excluding U01. Therefore, participant-level estimates and exploratory associations should be interpreted as preliminary and hypothesis generating rather than as definitive evidence.

Fifth, there are limitations inherent to the language models themselves when using BERT- or LLM-based approaches. Because these models are not fully transparent regarding which linguistic features drive their predictions, it is difficult to identify the causes of failure or ensure construct-level validity. In this study, we did not conduct a detailed evaluation of prompt design and limited the analysis to a simple prompt. Therefore, even when correlations with self-reports are observed, the extracted signals may partly reflect model-specific biases, and careful robustness testing is required.

Sixth, in the BERT-based evaluation, the company A training dataset and the company B external test dataset differed in both data collection timing and duration, which may have affected model performance. Because this study used existing workplace datasets, we could not separate the effects of company context from seasonal or temporal effects. In contrast, the Llama model was evaluated in a zero-shot setting and was not trained on the company A dataset. Future studies should collect data from multiple companies during the same or comparable periods and examine whether seasonality and differences in observation period affect model performance.

Finally, it should be emphasized that the target construct addressed in this study was work-related happiness derived from workplace diary texts (referred to as “workday happiness”) and that it should be distinguished from general happiness or broader subjective happiness.

### Conclusions

Measuring workday happiness as a determinant of well-being is increasingly recognized as crucial. While previous studies have used free-text descriptions to estimate happiness on a broad scale, limited research has focused on tracking individual fluctuations in workday happiness over time owing to the challenges associated with longitudinal data collection. This study addresses this issue by obtaining longitudinal data from two workplaces over 2 and 6 months. Subsequently, the data were used to construct a workday happiness estimation model and assess individual workday happiness levels. Evaluation of model performance using correlation coefficients showed variability in the correlation values among individuals. Although the sample size of 11 participants limits the generalizability of the findings, this study provides preliminary evidence supporting the feasibility of the proposed approach. Our findings highlight the potential of longitudinal, text-based approaches to capture the dynamic nature of workday happiness and support the design of interventions that foster healthier and more sustainable working lives.

## Supplementary material

10.2196/99992 Multimedia Appendix 1 Supplementary figures and tables on diary characteristics, model performance, and error-based metrics.

## Abbreviations

BERT: bidirectional encoder representations from transformers
LLM: large language model
MAE: mean absolute error
RMSE: root mean squared error
