# Supplementary material for: Estimating Longitudinal Changes in Workday Happiness From Daily Report Texts Among Employees: Retrospective Feasibility Study
Source: JMIR Form Res. 2026 Aug 25;10:e99992. doi: 10.2196/99992 (PMC13505971; doi:10.2196/99992)
Supplement: Supplementary file 1 [file formative-v10-e99992-s001.docx]

## **Multimedia Appendix**

Figure 3. Relationship between average sentence length and model performance. Panels show results for the BERT-based model and the locally deployed Llama model using scatterplots and median-split boxplots. U01 was excluded because model performance could not be calculated.

Figure 4. Relationship between lexical diversity and model performance for the BERT-based model and the locally deployed Llama model. Lexical diversity was measured using Self-BLEU, with higher values indicating lower lexical diversity. Scatterplots show participant-level associations, and boxplots compare below-median and above-median performance groups. U01 was excluded because model performance could not be calculated.

Table 4. Mean absolute error and root mean squared error for participant-level workday happiness estimation. MAE and RMSE were calculated for each participant and each model using the original 0-10 workday happiness scale.

| User ID | Number of data | BERT r (r²) | BERT P | BERT MAE | BERT RMSE | Llama r (r²) | Llama P | Llama MAE | Llama RMSE |
| --- | --- | --- | --- | --- | --- | --- | --- | --- | --- |
| U01 | 18 | - | - | 2.57 | 2.91 | - | - | 4.39 | 4.64 |
| U02 | 35 | 0.65 (0.42) | <.001 | 1.04 | 1.25 | 0.64 (0.41) | <.001 | 1.43 | 1.69 |
| U03 | 48 | -0.06 (0.00) | .701 | 1.54 | 1.86 | -0.05 (0.00) | .724 | 2.67 | 3.18 |
| U04 | 75 | 0.59 (0.34) | <.001 | 1.29 | 1.70 | 0.81 (0.66) | <.001 | 0.76 | 1.06 |
| U05 | 53 | 0.40 (0.16) | .003 | 2.78 | 3.50 | 0.72 (0.51) | <.001 | 1.49 | 1.91 |
| U06 | 61 | 0.40 (0.16) | .002 | 1.40 | 1.70 | 0.40 (0.16) | .002 | 2.43 | 2.79 |
| U07 | 87 | 0.63 (0.40) | <.001 | 1.35 | 1.63 | 0.76 (0.58) | <.001 | 0.91 | 1.23 |
| U08 | 66 | 0.63 (0.40) | <.001 | 1.22 | 1.56 | 0.64 (0.41) | <.001 | 1.67 | 2.04 |
| U09 | 47 | 0.49 (0.24) | <.001 | 1.25 | 1.49 | 0.39 (0.15) | .006 | 1.06 | 1.40 |
| U10 | 91 | 0.55 (0.31) | <.001 | 1.13 | 1.45 | 0.63 (0.40) | <.001 | 1.01 | 1.30 |
| U11 | 71 | 0.59 (0.35) | <.001 | 1.18 | 1.54 | 0.72 (0.51) | <.001 | 1.03 | 1.48 |
